# Translation and Validation of the Persian Version of the Rotator Cuff Quality of Life Questionnaire (RC‐QOL): A Cross‐Cultural Adaptation Study

**DOI:** 10.1002/hsr2.70912

**Published:** 2025-07-14

**Authors:** Masoud Gharib, Mehran Razavipour, Marziye Moradi‐Abbasabadi, Ebrahim Nasiri

**Affiliations:** ^1^ Department of Rehabilitation Sciences, School of Allied Medical Sciences Mazandaran University of Medical Sciences Sari Iran; ^2^ Orthopedic Research Center Mazandaran University of Medical Science Sari Iran; ^3^ Department of Anesthesiology, Operating Room, School of Allied Medical Sciences Mazandaran University of Medical Sciences Sari Iran

**Keywords:** pain, quality of life, rotator cuff injury, shoulder

## Abstract

**Background and Aims:**

Rotator cuff injuries have a significant impact on shoulder function and life quality. The Rotator Cuff Quality of Life (RC‐QOL) questionnaire is a validated tool designed to assess this impact. This study aimed to translate the RC‐QOL into Persian, adapting it culturally, and evaluate its psychometric properties in Persian‐speaking patients.

**Method:**

The Persian version of the RC‐QOL was developed through a rigorous translation and cultural adaptation process, including forward translation, expert review, back‐translation, and pilot testing. The final version underwent thorough content and face validity checks. Internal consistency was assessed using Cronbach'*α*, test‐retest reliability was evaluated using the intraclass correlation coefficient (ICC), construct validity was examined using confirmatory factor analysis, and convergent validity was assessed by correlating the RC‐QOL with the DASH and WOSI questionnaires.

**Results:**

Among the 176 Persian‐speaking participants, the average age was 38.01 ± 9.36 years. The Persian RC‐QOL displayed outstanding internal consistency, with Cronbach'*α* values ranging from 0.84 to 0.88, and strong test‐retest reliability, with ICC values between 0.82 and 0.88. Convergent validity was affirmed by significant correlations with DASH and WOSI questionnaires (ranging from −0.863 to −0.895, *p* < 0.001). Construct validity was confirmed using confirmatory factor analysis, with favorable fit indices: RMSEA = 0.04 and CFI = 0.97.

**Conclusion:**

The Persian version of RC‐QOL proves to be a reliable and valid tool for evaluating the quality of life in Iranian patients with rotator cuff injuries. Its successful cross‐cultural adaptation enhances its applicability in Iranian clinical and research domains, potentially extending to other Persian‐speaking regions.

## 1 Introduction

The shoulder pain is a common problem in the musculoskeletal pathology area [1] and can be regarded as the third most frequent complaint among primary care and physical therapy consultations [2, 3]. Rotator cuff injury is one of the most frequently mentioned pathological changes responsible for the incidence of this type of complaint [4]. The onset of shoulder pain can be based on various causes, including environmental and psychosocial ones [5, 6].

Age, therefore, plays a more pronounced role in the development of rotator cuff injuries, for the advancement of age and stress‐related strains can present themselves as incipient symptoms of such injuries [7]. Rotator cuff injury can have wide effects on the functioning of the shoulder joints and the performance of the upper limb [8]. The nature of this injury can generally affect the health status and quality of life of the concerned patients [9], impacting sleep, range of motion, and muscle strength [10, 11].

The outcomes of such injuries are interruptions in daily activities, leisure time, and work, very economically and socially, with great emotional consequences [12]. Various tools and tests are put into use during the assessment of the results of treatments meted on rotator cuff injuries [9]. Because tools such as the DASH and WOSI differ in content and form, how surgeons and their patients assess treatment outcomes may differ [13].

The RC‐QOL questionnaire is an important tool for assessing the impact of rotator cuff pathology on quality of life. This questionnaire was first introduced in the year 2000 for the evaluation of rotator cuff tears [14]. It was developed with an initial version with 55 items, and after several studies, this was reduced to 21 items. Based on the current questionnaire, which is proposed as a visual analogue scale ranging from 0 to 100, the scores are between 0 and 100 [14]. The RC‐QOL questionnaire consists of five subscales: Physical Symptoms with 16 items, Sports and Recreation with 4 items, Work with 4 items, Lifestyle with 5 items, and Emotions with 5 items. Currently, this questionnaire has been translated into German [15], Turkish [16], Italian [17], Chinese [18], Spanish [12], and Arabic languages [19]; it also has given suitable psychometric results related to reliability and validity for the mentioned languages.

So far, no version of the RC‐QOL questionnaire has been adapted to the Persian culture. Thus, a translation and cultural adaptation into Persian could be a very useful tool for health professionals in assessing quality of life among Persian‐speaking patients suffering from shoulder pain due to rotator cuff dysfunction.

The current study is aimed at translating and culturally adapting the RC‐QOL questionnaire in Persian through descriptive and observational methods. The secondary aim was to validate the reliability and validity of the Persian RC‐QOL questionnaire in Iranian Persian‐speaking patients suffering from rotator cuff injuries living in Iran.

## 2 Materials and Methods

### 2.1 Translation and Cultural Adaptation

Translation and transcultural adaptation of the RC‐QOL questionnaire into Persian was performed based on guidelines described by Beaton et al. [20]. First, two forward translations of the original questionnaire were performed by two Persian‐speaking Iranian translators, both fluent in English. One translator had a physiotherapy background, while the other was an occupational therapist. Then, the two translations were reviewed and compared simultaneously, based on which the final consensus version was prepared.

This version was, thus, reviewed for semantic, linguistic, and conceptual equivalence by a panel of experts and revised as needed.

Cultural adaptations included modifying sports examples (“baseball” replaced with “volleyball”) and colloquial phrases (“heavy lifting” adjusted to “carrying bulky objects”, Then, the final Persian version was back‐translated into English by two native English teachers. It was then approved after confirmation from the original questionnaire developer.

During content validity assessment, it was tested in a pilot study in 15 Persian‐speaking participants. After confirmation of content validity, the questionnaire was used for psychometric analysis.

### 2.2 Content and Face Validity

**Figure 1:**
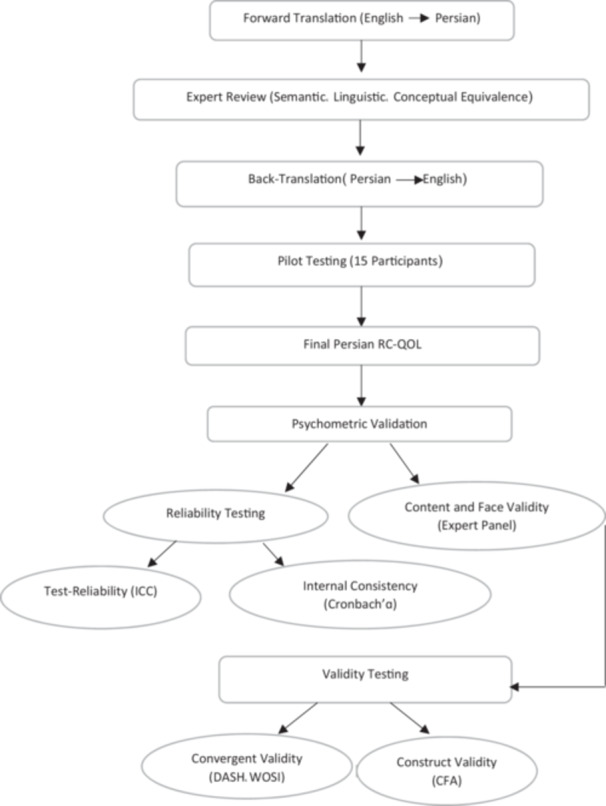
Flowchart of the translation and validation process.

Content and face validity were determined based on the feedback of 10 persons, in the form of three orthopedic specialists, five physiotherapists, and two occupational therapists. Secondly, the facility of readability of the scale items and instructions and face validity assessment was taken by three expert panels. Furthermore, experts were asked for clarity indices‐clear ‐unclear rating to further determine how effectively the questionnaire was presented. After that, the questionnaire was given to 10 experts who rated each item on the validity or relevance of content for its content equivalence. The following scale was used: 1 = not relevant, 2 = cannot assess relevance, 3 = relevant with minor modification, and 4 = very relevant and succinct. After final revisions based on expert panel feedback, the pre‐final version was converted to the final version (Figure 1).

### 2.3 Participants

Participants were recruited from Imam Khomeini Hospital in Sari, Iran, (2021–2023) and screened using MRI to confirm rotator cuff injuries. Participants completed the questionnaire in person during their clinic visits. All participants gave written informed consent, and the study was conducted following the ethical code of IR.MAZUMS.REC.1399.7164. Participants with either glenohumeral osteoarthritis or rotator cuff tear arthropathy, adhesive capsulitis, bone lesions, shoulder infections, glen humeral instability, and labral lesions were excluded. Test‐retest intervals were strictly 7 days (± 2 days). Participants outside this window (e.g., day 10) were excluded. MRI confirmation was mandatory for rotator cuff tear diagnosis; patients without MRI were excluded.

### 2.4 Measurement

Then, all subjects completed the RC‐QOL questionnaire in the Persian language. Convergent validity was measured using the Disabilities of the Arm, Shoulder, and Hand (DASH) [21] questionnaire and the Western Ontario Shoulder Instability Index (WOSI) questionnaire [22].

As designed by Hollinshead et al., the original RC‐QOL questionnaire consisted of 34 items within five domains, each measuring a different aspect of quality of life: Physical Symptoms (16 items), Sports and Recreation (4 items), Work (4 items), Lifestyle (5 items), and Emotions (5 items). Scoring is based on a VAS from 0 to 100, with higher scores reflecting better quality of life [14].

The DASH questionnaire is made up of 30 items, with two optional 4‐item modules that permit the assessment of the upper limb concerning functionality for several activities performed daily, in sports, leisure, and work. Thus, the variation in scores goes from 1 to 5, according to increasing symptoms. The total scores vary from 30 to 150 [21].

The WOSI questionnaire is made up of four domains: Physical Symptoms, 10 items; Sports/Recreation/Work, 4 items; Lifestyle, 4 items; Emotions, 3 items. Items are scored on a VAS from 0 to 100 and summed to provide a total score between 0 and 2100, with higher scores indicating worse shoulder function [22]. DASH and WOSI were chosen because they are widely used and validated tools for assessing shoulder function and disability.

Based on Kline's criteria, a sample size of 5–10 participants per item was required, resulting in a target sample size of 176 participants [23]. In the original RC‐QOL validation [14], we hypothesized strong negative correlations (*ρ* > 0.70) between Persian RC‐QOL and DASH/WOSI, as all tools assess shoulder‐related disability. Statistical analysis was performed using SPSS software, version 22.0 (IBM, Armonk, NY, USA).

Descriptive statistics were performed to characterize the participants. Internal consistency was assessed by the Cronbach's alpha coefficient interpreted as: < 0.7 low internal consistency; between 0.7 and 0.8 moderate consistency and > 0.8 high internal consistency [24].

The estimated test‐retest reliability through the ICC (two‐way random effects model) with the interval of 7 days was computed for each separate item, the set of items, and the five sections of the questionnaire. In case the ICC is above 0.8, the scale has good reproducibility.

The convergent validity of the RC‐QOL questionnaire with the questionnaires DASH and WOSI was tested by Spearman's correlation coefficient. Normality was assessed using Shapiro–Wilk tests and visual inspection (Q‐Q plots) before analysis. Since the data violated normality assumptions, non‐parametric Spearman's correlation was used. In all statistical calculations, the level *p* ≤ 0.05 was considered significant.

Construct validity was assessed using confirmatory factor analysis (CFA) to evaluate whether the Persian RC‐QOL adhered to the original five‐factor structure (physical symptoms, sports/recreation, work, lifestyle, and emotions). While we did not explicitly follow the COSMIN guidelines, our approach to validity testing was informed by its principles, including predefined hypotheses about the expected relationships between the Persian RC‐QOL and other validated questionnaires (DASH and WOSI) [25]. The analysis was conducted using AMOS software (version 22.0) with maximum likelihood estimation. Model fit was evaluated using the root mean square error of approximation (RMSEA), comparative fit index (CFI), and standardized root mean square residual (SRMR). A good model fit was indicated by RMSEA ≤ 0.05, CFI ≥ 0.95, and SRMR ≤ 0.08. Factor loadings were examined to ensure they were statistically significant (*p* < 0.05) and exceeded the threshold of 0.40. [26, 27, 28].

Floor and ceiling effects were also evaluated by calculating the number of participants achieving the lowest available (0) and highest available (100) scores on every RC‐QOL subscale as well as on the composite measure. According to Terwee et al. [29], greater than 15% of the effects were considered clinically relevant because they might reflect some limitations on the tool's discriminative capability among severity grades. This examination was done against the same database used for other psychometric verifications.

## 3 Results

The sample for this study included 176 participants, all of whom were native Persian speakers. The participants ranged in age from 19 to 57 years (38.01 ± 9.36 years). There were 46.02% women and 53.98% men (Table 1). Their average Body Mass Index (BMI) was 27.96 ± 3.3. Of the 176 participants, 62.5% had rotator cuff involvement on the right side and 37.5% on the left side.

**Table 1:** Demographic characteristics of participants.

Variables	Mean ± SD
Age (Year)	38.01 ± 9.36
Weight (Kg)	79.61 ± 10.86
Height (cm)	169.04 ± 4.32
BMI (kg/m^2^)	27.96 ± 3.3
Gender	*N* (%)
Female	81 (46.02)
Male	95 (53.98)
Affected side	*N* (%)
Right side	110 (62.5)
Left side	66 (37.5)

Abbreviation: BMI, body mass index.

### 3.1 Statistical Correlations

The data analysis revealed the following correlations between the RC‐QOL subscales and the WOSI: Symptoms, Work, Sports, Lifestyle, and Social/Emotional (*ρ *= −0.862 to −0.893; *p*< 0.001) and an overall score of (*ρ *= −0.863 to −0.893; *p*< 0.001) (Table 2). Furthermore, we also assessed the relationship between the RC‐QOL questionnaire and the DASH questionnaire, reporting correlations of Symptoms, Work, Sports, Lifestyle, and Social/Emotional (*ρ *= −0.872 to −0.895; *p*< 0.001) (Table 3).

**Table 2:** Spearman correlation between RC‐QOL and WOSI.

RC‐QOL	WOSI				
	Emotion	Lifestyle	Sports/recreation/work	Physical symptoms	Total WOSI
Symptoms	−0.862* (−0.88, −0.84)	−0.869* (−0.89, −0.85)	−0.853* (−0.87, −0.83)	−0.864* (−0.89, −0.83)	−0.864* (−0.89, −0.83)
Work	−0.881* (−0.90, −0.86)	−0.871* (−0.89, −0.85)	−0.869* (−0.89, −0.85)	−0.883* (−0.90, −0.86)	−0.893* (−0.91, −0.87)
Sports	−0.863* (−0.88, −0.84)	−0.861* (−0.88, −0.84)	−0.872* (−0.89, −0.85)	−0.860* (−0.88, −0.84)	−0.884* (−0.90, −0.86)
Lifestyle	−0.892* (−0.91, −0.87)	−0.874* (−0.89, −0.85)	−0.850* (−0.87, −0.83)	−0.868* (−0.89, −0.85)	−0.893* (−0.91, −0.87)
Social/emotional	−0.863* (−0.88, −0.84)	−0.862* (−0.88, −0.84)	−0.874* (−0.89, −0.85)	−0.887* (−0.90, −0.87)	−0.871* (−0.89, −0.85)
Total score	−0.881* (−0.90, −0.86)	−0.870* (−0.89, −0.85)	−0.859* (−0.88, −0.84)	−0.869* (−0.89, −0.85)	−0.863* (−0.88, −0.84)

Abbreviations: RC‐QOL, rotator cuff quality of life; WOSI, Western Ontario Shoulder Instability Index

* *p*value < 0.001.

**Table 3:** Correlation between RC‐QOL and DASH.

RC‐QOL	DASH	95% Confidence interval (CI)	*p*
Symptoms	−0.895	(−0.91, −0.87)	*p* < 0.001
Work	−0.873	(−0.89, −0.85)	*p* < 0.001
Sports	−0.893	(−0.91, −0.87)	*p* < 0.001
Lifestyle	−0.872	(−0.89, −0.85)	*p* < 0.001
Social/emotional	−0.885	(−0.90, −0.86)	*p* < 0.001
Total score	−0.879	(−0.90, −0.86)	*p* < 0.001

Abbreviations: DASH, disabilities of the arm, shoulder, and hand; RC‐QOL, rotator cuff quality of life.

### 3.2 Reliability and Internal Consistency

The internal consistency of subscales of Symptoms, Work, Sports, Lifestyle, Social/Emotional, and Overall Score is as follows, with Cronbach's alpha coefficients of 0.88, 0.86, 0.85, 0.87, 0.84, and 0.85, respectively. The test‐retest reliability of the subscales ranged from ICC 0.82 to 0.88 (*p *< 0.001) for the Overall Score, ICC was 0.84 (*p *< 0.001) (Table 4).

**Table 4:** Test‐retest reliability and internal consistency (Cronbach's *α*) of the Persian RC‐QOL.

RC‐QOL	Cronbach's *α* (95% CI)	ICC (95% CI)	*p*
Symptoms	0.88 (0.85–0.91)	0.86 (0.83–0.91)	*p* < 0.001
Work	0.86 (0.83–0.89)	0.85 (0.79–0.89)	*p* < 0.001
Sports	0.85 (0.82–0.88)	0.88 (0.85–0.92)	*p* < 0.001
Lifestyle	0.87 (0.84–0.90)	0.83 (0.78–0.88)	*p* < 0.001
Social/emotional	0.84 (0.81–0.87)	0.82 (0.79–0.86)	*p* < 0.001
Total	0.85 (0.82–0.88)	0.84 (0.79–0.87)	*p* < 0.001

Abbreviations: CI, confidence interval; ICC, intraclass correlation coefficient; RC‐QOL, rotator cuff quality of life.

### 3.3 Construct Validity

The findings from the construct validity assessment demonstrated that the model was adequate. Specifically, the RMSEA, SRMR, CFI, NFI, and AGFI were found to be 0.04, 0.05, 0.97, 0.93, and 0.95, respectively, which indicates model validity, as shown in Table 5. Factor loadings for the Symptoms, Work, Sports, Lifestyle, Social/Emotional, and Overall Score scales ranged from 0.51 to 0.62. Furthermore, the model was considered statistically significant for the subscales as well as the overall score (*p* < 0.05).

**Table 5:** Construct validity analysis results.

Subscale	Load factor	RMSEA	SRMR	CFI	NFI	AGFI	*p*
Symptoms	0.51	0.03	0.06	0.92	0.97	0.91	*p* < 0.05
Work	0.62	0.04	0.02	0.92	0.91	0.95	*p* < 0.03
Sports	0.52	0.01	0.05	0.99	0.91	0.93	*p* < 0.05
Lifestyle	0.58	0.03	0.08	0.94	0.90	0.97	*p* < 0.05
Social/emotional	0.60	0.05	0.07	0.99	0.99	0.92	*p* < 0.05
Total	0.51	0.04	0.05	0.97	0.93	0.95	*p* < 0.04

Abbreviations: AGFI, adjusted goodness of fit indices; CFI, comparative fit index; NFI, normal fit index; RMSEA, root mean square error of approximation; SRMR, standardized root mean square residual.

Floor and ceiling effects were explored to measure response distribution extremes (Table 6). There were negligible effects (0–3.4%) in all subscales, well below the 15% cut point for clinical significance. Most ceiling effect was noted in lifestyle items (3.4%) and there was a 1.7% floor effect in Sports/Recreation.

**Table 6:** Floor and ceiling effects of the Persian RC‐QOL (*N* = 176).

Subscale	Floor (% at 0)	Ceiling (% at 100)
Symptoms	0.6% (1/176)	2.8% (5/176)
Work	1.1% (2/176)	1.7% (3/176)
Sports/recreation	1.7% (3/176)	0.6% (1/176)
Lifestyle	0% (0/176)	3.4% (6/176)
Social/emotional	1.1% (2/176)	1.1% (2/176)
Total score	0% (0/176)	0.6% (1/176)

## 4 Discussion

This study aimed to translate and culturally adapt the RC‐QOL questionnaire into Persian and evaluate its psychometric properties in Persian‐speaking patients with rotator cuff injuries. Through a meticulous validation process, we sought to validate the reliability and validity of the Persian version of RC‐QOL in Persian‐speaking patients suffering from rotator cuff injuries. The primary discovery of this study indicates that the RC‐QOL questionnaire holds validity among Iranian patients speaking Persian and dealing with rotator cuff ailments, displaying consistent reliability, internal coherence, and validity.

### 4.1 Translation and Cultural Adaptation

The translation process followed established guidelines by Beaton et al. [20], ensuring semantic, linguistic, and conceptual equivalence. The rigorous process, including forward and back‐translation, expert review, and pilot testing, resulted in a Persian version that retained the original questionnaire's intent while being culturally appropriate for Iranian patients. This approach aligns with previous adaptations of the RC‐QOL in other languages, such as Spanish, Turkish, and Arabic, which also emphasized the importance of cultural relevance and conceptual equivalence [12, 16, 18, 19].

### 4.2 Reliability and Internal Consistency

The Persian RC‐QOL demonstrated excellent reliability, with high internal consistency (Cronbach's *α* ranging from 0.84 to 0.88) and strong test‐retest reliability (ICC values between 0.82 and 0.88). These findings are consistent with previous adaptations of the RC‐QOL, such as the Spanish version (Cronbach's *α*: 0.85–0.90) [12] and the Turkish version (ICC: 0.82–0.89) [16]. The high internal consistency indicates that the items within each subscale measure the same underlying construct, while the strong test‐retest reliability suggests that the questionnaire produces stable results over time. These results confirm that the Persian RC‐QOL is a reliable tool for assessing quality of life in patients with rotator cuff injuries.

### 4.3 Construct and Convergent Validity

Construct validity was supported by confirmatory factor analysis, which showed excellent model fit (RMSEA = 0.04, CFI = 0.97). The factor loadings for all subscales were statistically significant and exceeded the threshold of 0.40, confirming that the Persian RC‐QOL measures the intended constructs effectively. Convergent validity was assessed by correlating the Persian RC‐QOL with the DASH and WOSI questionnaires. We predefined hypotheses based on the original RC‐QOL validation studies, expecting strong negative correlations (*r* > 0.70) between the RC‐QOL and DASH/WOSI. These hypotheses were confirmed, with correlations ranging from −0.863 to −0.895 (*p* < 0.001), exceeding the 75% threshold recommended by COSMIN guidelines. While we did not explicitly follow COSMIN guidelines, our approach aligns with its core principles, and the results demonstrate robust construct validity. The negative correlations are expected, as higher scores on the RC‐QOL indicate better quality of life, while higher scores on the DASH and WOSI indicate greater disability and poorer shoulder function. These findings are consistent with other linguistic versions of the RC‐QOL, such as the Spanish [12] and Arabic [19] adaptations, which also reported strong correlations with similar instruments.

The Persian RC‐QOL had minimal floor (0–1.7%) and ceiling effects (0.6–3.4%) on all subscales, much lower than the 15% clinical cutoff [29]. The slightly higher ceiling effect on Lifestyle items (3.4%) may be due to cultural adaptations in activity questions, while the floor effect of the Sports subscale (1.7%) is congruent with expected activity restriction in rotator cuff pathology. These findings support the instrument's sensitivity to detect change in all severity levels, which surpasses the initial English version [14].

### 4.4 Clinical Implications

The Persian RC‐QOL fills a significant gap in the assessment of rotator cuff injuries among Persian‐speaking populations. Its successful adaptation and validation provide clinicians and researchers with a reliable and valid tool to evaluate the impact of rotator cuff injuries on patients' quality of life. This questionnaire can be used in clinical practice to monitor treatment outcomes and in research to compare interventions across different populations. The availability of a culturally adapted version of the RC‐QOL also facilitates cross‐cultural comparisons and enhances the generalizability of findings in international studies.

### 4.5 Limitations and Future Directions

This study has some limitations. First, the sample was recruited from a single region in Iran, which may limit generalizability to other Persian‐speaking populations. Second, the study did not assess the minimal clinically important difference (MCID), minimal detectable change (MDC), or floor and ceiling effects, which are important for interpreting score changes over time. Additionally, detailed information about the type of rotator cuff injury (e.g., isolated vs. multiple, specific tendons involved) and patient characteristics (e.g., educational background, occupation) was not collected. Future studies should validate the Persian RC‐QOL in diverse settings, address these psychometric properties, and include more detailed patient data to enhance clinical utility and generalizability.

## 5 Conclusion

The Persian version of the RC‐QOL demonstrates excellent psychometric properties, including high reliability, strong validity, and cultural relevance. It is a valuable tool for assessing the quality of life in Persian‐speaking patients with rotator cuff injuries and can be used in both clinical and research settings. This study contributes to the growing body of evidence supporting the cross‐cultural applicability of the RC‐QOL and underscores its potential as a standardized assessment tool for rotator cuff pathology.

## Author Contributions

**Masoud Gharib:** conceptualization, investigation, funding acquisition, writing – original draft, writing – review and editing, visualization, validation, methodology, software, formal analysis, project administration, resources, supervision, and data curation. **Mehran Razavipour:** conceptualization, investigation, funding acquisition, writing – original draft, writing – review and editing, visualization, validation, methodology, software, formal analysis, project administration, resources, supervision, and data curation. **Marziye Moradi‐Abbasabadi:** resources, supervision, data curation, project administration, formal analysis, software, visualization, writing – review and editing, validation, methodology, conceptualization, investigation, funding acquisition, and writing – original draft. **Ebrahim Nasiri:** conceptualization, investigation, funding acquisition, writing – original draft, writing – review and editing, visualization, methodology, validation, project administration, formal analysis, software, resources, supervision, and data curation.

## Conflicts of Interest

The authors declare no conflicts of interest.

## Transparency Statement

The corresponding author, Masoud Gharib, affirms that this manuscript is an honest, accurate, and transparent account of the study being reported; that no important aspects of the study have been omitted; and that any discrepancies from the study as planned (and, if relevant, registered) have been explained.

## Data Availability

The data supporting this study are available from the corresponding author upon request.
